# Light Driven CO_2_ Fixation by Using Cyanobacterial Photosystem I and NADPH-Dependent Formate Dehydrogenase

**DOI:** 10.1371/journal.pone.0071581

**Published:** 2013-08-06

**Authors:** Masaki Ihara, Yusuke Kawano, Miho Urano, Ayako Okabe

**Affiliations:** 1 Faculty of Agriculture, Shinshu University, Minamiminowa, Nagano, Japan; 2 Precursory Research for Embryonic Science and Technology, Japan Science and Technology Agency, Honcho, Kawaguchi, Saitama, Japan; University of Hyderabad, India

## Abstract

The ultimate goal of this research is to construct a new direct CO_2_ fixation system using photosystems in living algae. Here, we report light-driven formate production from CO_2_ by using cyanobacterial photosystem I (PS I). Formate, a chemical hydrogen carrier and important industrial material, can be produced from CO_2_ by using the reducing power and the catalytic function of formate dehydrogenase (FDH). We created a bacterial FDH mutant that experimentally switched the cofactor specificity from NADH to NADPH, and combined it with an *in vitro*-reconstituted cyanobacterial light-driven NADPH production system consisting of PS I, ferredoxin (Fd), and ferredoxin-NADP^+^-reductase (FNR). Consequently, light-dependent formate production under a CO_2_ atmosphere was successfully achieved. In addition, we introduced the NADPH-dependent FDH mutant into heterocysts of the cyanobacterium *Anabaena* sp. PCC 7120 and demonstrated an increased formate concentration in the cells. These results provide a new possibility for photo-biological CO_2_ fixation.

## Introduction

Formate is an important energy carrier in the bacterial kingdom. Biological formate is produced by the degradation of pyruvate, amino acids, l-(+)-tartaric acid, oxalate, and hypoxanthine [1,2]. It can also be produced by the direct reduction of CO_2_ catalyzed by formate dehydrogenase (FDH), and this approach is increasingly gaining attention [3].

FDHs consist of several groups of enzymes that vary significantly in their quaternary structure, occurrence, and type of prosthetic groups. One such group is the NADH-dependent FDH (EC1.2.1.2) (NADH-FDH), which catalyzes the oxidation of formate to CO_2_ and the reduction of CO_2_ to formate coupled with the oxidation-reduction of NAD^+^/NADH (HCOO^-^ + NAD^+^ ↔ NADH + CO_2_) [4]. NADH-FDH has previously been applied to light-driven formate production systems by using Zn-porphyrin or TiO_2_ particles as a photosensitizer [5–7]. In these systems, electrons are supplied by photosensitizers and transported via mediators such as methyl viologen, to diaphorase reducing NAD^+^ to NADH. The NADH-FDH then catalyzes the CO_2_ reduction by using the resulting NADH to produce formate.

**Figure 1:**
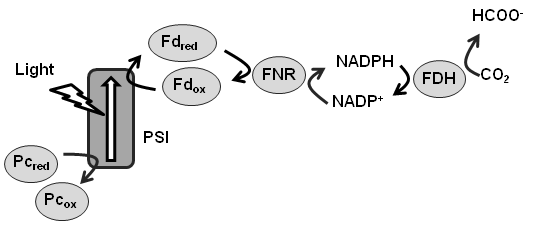
The proposed system for light-driven formate production.

Based on the results of previous studies, we designed a new biological system (Figure 1). In our system, the ability of cyanobacteria, algae, and plants to effectively produce NADPH using a photosynthetic light reaction [8,9] was taken as the basis for the design, in order to produce formate by using NADPH as a substrate for FDH. All the components are biologically producible molecules and can, therefore, be regenerated within the cell, enabling the creation of a sustainable formate production mechanism.

In oxygenic photosynthesis, photochemical reactions are carried out in 2 separate photosystems. Photosystem II (PS II) catalyzes the anodic half-cell reaction: H_2_O + 2hν → 1/2O_2_ + 2e^-^ + 2H^+^, whereas photosystem I (PS I) catalyzes the cathodic half-cell reaction: oxidized Fd (Fd_ox_) + reduced plastocyanin (PC_red_) + hν → reduced Fd (Fd_red_) + oxidized PC (PC_ox_). Fd_red_ then transfers electrons to NADP^+^ via ferredoxin-NADP^+^-reductase (FNR), thereby reducing it to NADPH: 2Fd_red_ + H^+^ + NADP^+^ → 2Fd_ox_ + NADPH. NADPH can be used as a reducing agent in various biosynthetic processes, including CO_2_ fixation via the Calvin–Benson cycle. However, direct formate production by using NADPH and CO_2_ does not progress in oxygen-evolving phototrophs because of the oxygen-sensitivity of FDH.

To create a light-driven formate production system *in vivo*, we focused on cyanobacterial heterocysts, which carry out a strictly anaerobic reaction, i.e., nitrogen fixation [10]. Heterocysts differentiate from vegetative cells, which carry out oxygen-evolving photosynthesis, and are deficient in PS II activity. Their internal environment is, therefore, micro-oxic and oxygen-sensitive nitrogenase is expressed in the cells. The electrons necessary for the nitrogen fixation are supplied from neighboring vegetative cells in the form of sucrose. Sucrose is degraded into CO_2_, protons, and electrons, and the electrons are photo-excited by PS I and transferred to nitrogenase to reduce nitrogen [10]. The micro-oxic and reductive environment makes heterocysts an ideal reaction vessel for light-driven formate production by FDH.

We first created NADPH-dependent FDH (NADPH-FDH) because all the known NADH-FDHs are specific for NADH. In a previous study, site-saturation mutagenesis at the amino acid residues determining the cofactor specificity of NADH-FDH were examined and some mutants, in which the cofactor preference in the degradation of formate was shifted from NAD^+^ to NADP^+^, were found [11]. However, these studies did not report any data on the productivity of formate and its stability. Here, we prepared several mutants of FDHs from *Pseudomonas* sp. 101 ( *Pseudomonas* ), *Candida* *boidinii* (Candida), potato, *Arabidopsis thaliana* ( *Arabidopsis* ), and *Thiobacillus* sp. KNK65MA ( *Thiobacillus* ), and investigated their formate productivity from NADPH and CO_2_. We then verified our formate production system *in vitro* and in heterocysts by using the NADPH-FDH with the highest formate productivity.

## Materials and Methods

### Bacterial strains and Culture conditions

*Escherichia coli* (*E. coli*) DH5α (RBC Bioscience, New Taipei City, Taiwan) and *E. coli* BL21 (DE3) (Merck, Rahway, New Jersey) were used for the genetic manipulation and expression of recombinant FDH, respectively. For protein expression, 0.5 mM (as a final concentration) of isopropyl-l-d-thiogalactopyranoside (IPTG; Nacalai tesque, Kyoto, Japan) was added to each Luria-Bertani (LB) medium. The filamentous heterocystous cyanobacterium *Nostoc* sp. strain PCC 7120 (also called *Anabaena* sp. strain PCC 7120) (hereinafter, *Anabaena* ) was purchased from the Pasteur Culture Collection of *Cyanobacteria* (France). Anabaena and the transformed cells were grown at 30^◦^C under a light intensity of 30 µE∙m^-2^∙s^-1^ in the BG11 medium as previously described [12]. Liquid cultures were bubbled with air. For nitrogen deprivation experiments, cells grown in the BG11 medium until they reached an OD_600_ of 0.4–0.5 were washed with the nitrogen-free medium (BG11_0_) and then re-suspended in the BG11_0_ medium. Neomycin was added to the medium at a final concentration of 30 µg/mL, when required.

### Plasmids

The synthesized genes coding for FDHs from *Pseudomonas* , *Candida* , potato, *Arabidopsis* , and *Thiobacillus* were subcloned using *Nde*I and *Not*I sites in pET22b (Merck) as C-terminal His-tagged proteins. According to previous studies [11], the amino acid residues determining the cofactor specificity of NADH-FDH were suggested to be at 222 and 224 in *Pseudomonas* , 197 and 199 in potato, 227 and 229 in *Arabidopsis* , and 222 and 224 in *Thiobacillus* . The nucleotides corresponding to the above pairs of amino acids were substituted with those corresponding to Glu/Ser/Ala/Gln and Asn/His, respectively, by means of PCR site-directed mutagenesis using mixed primers, 5′-GTTCATCTGCACTATACC (C or G or T) (A or C) GCGT(A or C) AC CGCCTGCCGGAAAGCGTG-3′ for *Pseudomonas* , 5′-CTGCAATCTGCTGTACCAC (C or G or T)(A or C)GCGT(A or C) A CAAAATGGATTCTGAACTG-3′ for potato, 5′- GTTGCAACCTGCTGTACCAT (C or G or T)(A or C) GCGT(A or C) A CCAGATGGCACCGGAACTG -3′ for *Arabidopsis* , and 5′-GTTAAACTGCATTATACC (C or G or T)(A or C) GCGT(A or C) A CCGTCTGCCGGAAGCAGTG-3′for *Thiobacillus* , and their complementary primers (underlines indicate codons corresponding to amino acid residues determining the cofactor specificity). The introduction of mutations into *Candida* gene was performed using non-mixed primers, 5′-CGAAAGAACTGCTGTATTACC A G C G T A A TGCCCTGCCGAAAGAAGCAG-3′ and its complementary primer (underlines indicate codons corresponding to mutation sites (Asp195 → Gln, Tyr196→Arg and Gln197→Asn)).

The DNA fragments coding for plastocyanin (Pc), ferredoxin (Fd) and ferredoxin–NADP^+^ reductase (FNR) were amplified from *Synechocystis sp.* PCC6803 genome using primer pairs; 5′-AGATATACATATGTCTAAAAAGTTTTTAAC-3′ and 5′-ATATAGTGCGGCCGCCTCAACGACAACTTTGCC-3′ for Pc, 5′-AGATATACATATGGCATCCTATACCGTTA-3′ and 5′-ATATAGTGCGGCCGCGTAGAGGTCTTCTTCTTTG-3′ for Fd, and 5′-AGATATACATATGTACAGTCCCGGTTAC-3′ and 5′-ATATAGTGCGGCCGCGTAGGTTTCCACGTGCCAG-3′ for FNR, respectively. The fragments were digested with *Nde*I and *Not*I, and inserted into pET22b treated with the same enzymes.

For the expression of NADPH-FDH in *Anabaena* heterocysts, we used a *hetR* promoter and a shuttle vector, pAM505, which contained replicons of *E. coli* and *Anabaena* [12,13]. A fragment coding for the NADPH-FDH gene was amplified from pET22b harboring a gene coding for a *Pseudomonas* FDH mutant, PsFDH[QN] (please see Results and Discussion), using primers, 5′-AAACTGGAGACTCATAATGGCGAAAGTGCTGTG-3′, and 5′-AATAATTGAGCTCTCAGTGGTGGTGGTGGTGGTGTTCGAGTGCGGCCGC-3′. The *hetR* promoter (approximately 1000 bp upstream of *hetR* coding region) was amplified from *Anabaena* genome according to Wang et al. [14]. The fragments were then digested and inserted into pAM505 using *Sal*I and *Sac*I sites, resulting in pAM505-fdh. Furthermore, the FNR gene was inserted into the downstream of the FDH, generating a vector for co-expression of FDH and FNR, pAM505-fdhfnr.

The helper plasmid pRL623, which harbors a *mob* gene and methylase genes [15] and a self-transmissible plasmid RP4 [16] were provided from Prof. Wolk (Michigan State University).

#### Introduction of the shuttle vector into *Anabaena*

Plasmid pAM505-fdh was first introduced into *E. coli* HB101 carrying a helper plasmid pRL623 [15]. The resultant transformed cells were mixed with *Anabaena* and *E. coli* J53 carrying a self-transmissible plasmid RP4 [16–18], and the exoconjugant *Anabaena* carrying pAM505-fdh ( *Anabaena* -fdh) was selected on a BG11 agar plate containing neomycin. For co-expression of PsFDH(QN) and FNR, we introduced pAM505-fdhfnr into *Anabaena* and isolated the exoconjugant *Anabaena* -fdhfnr similarly to that described above.

### Purification of proteins

The FDH proteins, Pc, Fd and FNR were purified under aerobic conditions using a His-Accept Ni-chelating resin (Nacalai tesque). The 50 mM Tris buffer (pH 7.6) containing 500 mM NaCl was used as binding and wash buffer. The binding buffer containing 500 mM imidazole was used for the elution of His-tagged proteins.

Purification of PS I was conducted essentially as previously described by Nakamoto and Hasegawa [19] except for the solubilization condition. To solubilize PS I from thylakoid membrane, the membrane was suspended in the buffer containing 1.6% dodecyl-β-d-maltoside, 50 mM Tris-HCl (pH8.0), 10 mM NaCl and 10% sucrose.

### SDS-PAGE and western blot analyses

For SDS-PAGE, gradient gels (5–20%, SuperSepTMAce, Wako, Osaka, Japan) and the Rapid Stain CBB Kit (Nacalai Tesque) were used, according to manufacturer’s instructions. For western blot analysis, proteins in SDS-PAGE gel were transferred onto polyvinylidene difluoride membrane (Immobilon, Millipore) for 1 h under 80 mA constant-current conditions. Blots were blocked with 0.5% (w/v) skim milk in TBST buffer (25 mM Tris-HCl pH7.4, 150 mM NaCl, 0.1% (v/v) Tween 20) and then incubated with a mouse biotin-conjugated anti-histidine-tag antibody (Penta-His Biotin Conjugate, Qiagen, Venlo, Netherlands) and Streptavidin-Horseradish Peroxidase conjugate (Prozyme, Hayward, California) diluted in blocking solution. Blots were washed with TBST buffer. Immunoreaction of HRP was carried out using Pierce Western Blotting Substrate (Thermo Scientific, Headquarters, Waltham, Massachusetts). The resulting chemiluminescence was detected using the ImageQuant LAS 4000 mini (GE Healthcare, Little Chalfont, United Kingdom).

### Assays

The formate degradation activities of the FDH proteins were measured spectroscopically in 2 mL of phosphate-buffered saline (PBS) containing 1 mM NADP^+^ or NAD^+^, 100 mM formate, 0.05% Triton X-100, and 5 µg/mL FDH. The reaction was initialized by the addition of a FDH sample and the change in the absorption of NADPH (or NADH) was monitored at 340 nm (the extinction coefficient is 6.22 mM^-1^cm^-1^). The analysis was performed in triplicate. The Michaelis-Menten parameters were determined from the NAD(P)^+^ dependence of initial rates of NAD(P) H production in PBS containing 0.05–2 mM NAD(P) D^+^, 500 mM formate, 0.05% Triton X-100, and 5 µg/mL FDH, and from the formate dependence obtained in the same conditions, except for the NAD(P) D^+^ and formate concentrations being constant (2 mM) and variable (10–500 mM), respectively.

The formate formation reaction was carried out under strictly anaerobic conditions. A tightly sealed branched flask was used with FDH placed in one arm and PBS, NADPH, and Triton X-100 in the other. The 2 solutions were degassed by repeated cycles of evacuation and argon gas purging, followed by charging with 100% CO_2_ for 1 min at the final cycle. The reaction was initiated by mixing the 2 solutions at 25° C. The final concentrations of FDH, NAD(P) H, and Triton X-100 were 1.7 mg/ml (38 µM), 1 mM, and 0.05%, respectively. The concentration of the dissolved CO_2_ in the buffer was measured using a CO_2_ sensor, CGP-31 (TOA DKK, Tokyo, Japan), and it was observed to be between 1.0 and 1.3 mg/ml. The concentration of formate was determined by withdrawing 100-µl aliquots from the solution without exposure to O_2_ at intervals of 60 min, and subsequently analyzing them with ion chromatography by using a HPLC system equipped with a Shim-pack SCR-102H column (300 mm length × 8.0 mm φ; mobile phase, 4 mM *p*-toluenesulfonic acid; column temperature, 40° C) and a conductivity detector, CDD-10A_VP_ (Shimadzu, Kyoto, Japan). The analysis was performed in triplicate.

Light-dependent formate production was performed in 3 mL PBS by using isolated PS I at a concentration of 100 µg chlorophyll/mL, 10 mM ascorbate, 10 µM PC, 2 µM Fd, 0.5 µM FNR, 1 mM NADP^+^, 4 mM NADPH, 2.5 mg/mL (56 µM) FDH mutant (PsFDH[QN]), 0.05% Triton X-100, and 10% sucrose. The solution containing all the components was degassed by repeating cycles of evacuation and argon gas purging, followed by charging with 100% CO_2_ for 1 min during the final cycle. The reaction was initiated by illuminating with visible light (<420 nm) at an intensity of 1000 µmol (photon) m^-2^ s^-1^ and monitored by sampling at regular intervals without exposure to O_2_. The analysis was performed in triplicate.

Intracellular formate levels were estimated using the extracts from *Anabaena* -WT, *Anabaena* -fdh and *Anabaena* -fdhfnr cell pellets. *Anabaena* -WT, *Anabaena* -fdh and *Anabaena* -fdhfnr were initially grown in BG11 medium by bubbling with air under a fluorescent lamp at an intensity of 15 µmol photon m^-2^ s^-1^, and the cells in the exponential growth phase (OD_700_ ~3) were washed and transferred into BG11_0_ medium and cultured for a further 36 h. The formation of heterocysts was confirmed microscopically. The media were then degassed and bubbled with air, air + 10% CO_2_, N_2_ + 10% CO_2_, or argon + 10% CO_2_, for 12 h under relatively strong light (150 µmol photon m^-2^ s^-1^). The cultures were transferred anaerobically to the centrifuge tubes in a globe box, centrifuged at 20,000 × g and lysed by grinding with glass beads in 0.1N HCl. The lysates were adjusted to pH 3, centrifuged, and subjected to HPLC analysis. The cultivation and analysis were performed in triplicate.

## Results and Discussion

### Conversion of the cofactor specificities of FDH mutants

To engineer NADH-FDHs, the FDH genes from *Pseudomonas* , *Candida* , potato, *Arabidopsis* , and *Thiobacillus* were subcloned into pET22b and mutated at the positions corresponding to the amino acid residues determining the cofactor specificity (i.e., 222 and 224 in *Pseudomonas* , 195–197 in *Candida* , 197 and 199 in potato, 227 and 229 in *Arabidopsis* , and 222 and 224 in *Thiobacillus* ). The resulting plasmids were transformed into *E. coli* BL21 (DE3). Cells were grown in LB medium at 37° C and induced with 0.5 mM IPTG. FDH mutants were expressed as a soluble and apo form and purified under aerobic conditions. SDS-PAGE analyses indicated that purified FDH samples from *Arabidopsis* and *Thiobacillus* contained additional bands at a slightly lower molecular weight besides an expected band, indicating the partial digestion during the purification; however, those from *Pseudomonas* , *Candida* and potato were purified to homogeneity.

Cofactor specificities of FDH mutants were first evaluated by measuring the NAD^+^ or NADP^+^ reduction rate. FDH mutants from *Arabidopsis* and *Thiobacillus* tended to aggregate and become inactivated during the reaction; however, those from *Pseudomonas* , *Candida* , and potato were relatively stable. Several mutants switched the cofactor specificity from NADH to NADPH (Table 1), and among them, *Pseudomonas* FDH mutations (222→Gln/224→Asn, PsFDH[QN]), *Candida* FDH mutant (195→Gln/196Arg/197→Asn, CbFDH[QRN]), *Arabidopsis* FDH mutant (227→Gln/197→Asn, AtFDH[QN]) and potato FDH mutant (195→Gln/197 → His, PoFDH[QH]) exhibited a relatively high NADP^+^ reduction activity. Using these selected mutants, we next determined the Michaelis-Menten parameters. As shown in Table 2, PsFDH[QN] and CbFDH[QRN] exhibited the highest V_max_ values compared with the other 2 mutants, AtFDH[QN] and PoFDH[QH]. PsFDH[QN] showed relatively low *K*_m_ values in both substrates, NADP and formate, implying a relatively small perturbation by the mutations. *K*_m_ values for NAD^+^ of the selected mutants, except for PsFDH[QN], could not be determined because of the low affinity for NAD^+^ in the concentration range examined (0–20 mM).

**Table 1:** Cofactor specificities of the WT and mutant FDH proteins.

	**NADP^+^ reduction** (µM/h^-1^)	**NAD^+^ reduction** (µM/h^-1^)	**NADP^+^/NAD^+^**
** *Pseudomonas* **			
WT (DH)	24.0 ± 1.7	1010 ± 44	0.024
EN	30.0 ± 0.5	417 ± 45	0.072
SN	279 ± 1	579 ± 30	0.48
SH	184 ± 2	429 ± 10	0.43
AN	141 ± 6	138 ± 2	1.0
AH	153 ± 2	203 ± 10	0.75
QN	293 ± 5	30.9 ± 4.2	9.5
QH	299 ± 13	31.0 ± 3.9	9.6
** *Candida* **			
WT (DYH)	16.8 ± 0.6	302 ± 4	0.056
QRN	265 ± 0.3	51.0 ± 9.6	5.2
** *Thiobacillus* **			
WT (DH)	18.9 ± 0.4	926 ± 6	0.020
SN	0.4 ± 0.2	3.9 ± 2.9	0.10
SH	3.0 ± 1.2	2.6 ± 1.2	1.15
AN	0 ± 0	3.1 ± 2.6	0
AH	0.2 ± 0.1	3.2 ± 2.6	0.063
QN	0.8 ± 0.1	0.3 ± 0.1	2.6
QH	0.6 ± 0.4	0.4 ± 0.2	1.5
** *Arabidopsis* **			
WT (DL)	1.3 ± 0.2	416 ± 61	0.0031
SN	6.8 ± 3.0	5.4 ± 4.0	1.3
SH	4.5 ± 2.6	4.9 ± 4.4	0.92
AN	5.8 ± 3.0	3.1 ± 2.3	1.9
AH	14.6 ± 1.7	3.3 ± 0.9	4.4
QN	3.4 ± 2.4	5.1 ± 1.6	0.67
QH	70.5 ± 13.9	4.2 ± 2.7	17
**Potato**			
WT (DL)	6.7 ± 0.2	803 ± 3	0.0083
EN	5.5 ± 1.5	112 ± 1	0.049
SN	53.1 ± 0.7	8.6 ± 3.1	6.2
SH	91.7 ± 5.5	75.5 ± 1.8	1.2
AH	116 ± 2	11.1 ± 3.8	10
QN	93.4 ± 1.4	3.7 ± 2.3	25.2
QH	149 ± 11	3.4 ± 1.0	44

FDHs were added up to a final concentration of 5 µg/mL into PBS containing 0.8 mM NAD^+^ or NADP^+^, 50 mM sodium formate, and 0.05% Triton X-100 at 30° C. NADH or NADPH production was monitored at 340 nm. All the measurements were performed in triplicate. The two-letter or three-letter descriptions in the left column indicate 2 or 3 one-letter codes of amino acid residue at the position of 222 and 224 in *Pseudomonas* , 195, 196 and 197 in *Candida* , 197 and 199 in potato, 227 and 229 in *Arabidopsis* , and 222 and 224 in *Thiobacillus* .

**Table 2:** Michaelis-Menten parameters of the mutant FDH proteins.

	**NADP^+^ reduction**			**NAD^+^ reduction**	
	V_max_	*K*_m_(NADP^+^)	*K*_m_(formate)	V_max_	*K*_m_(NAD^+^)
	(µM/h^-1^)	(mM)	(mM)	(µM/h^-1^)	(mM)
PsFDH[QN]	590 ± 20	0.35 ± 0.01	63 ± 3	33 ± 23	1.0 ± 0.6
CbFDH[QRN]	500 ± 200	0.18 ± 0.01	150 ± 28	ND	ND
AtFDH[QN]	260 ± 34	0.91 ± 0.07	96 ± 14	ND	ND
PoFDH[QH]	320 ± 19	0.62 ± 0.05	120 ±12	ND	ND

The reactions were carried out in PBS containing 0.05–2 mM NAD(P)^+^ 500 mM formate, 0.05% Triton X-100, and 5 µg/mL FDH or in the same conditions, except for the NAD(P) D^+^ and formate concentrations being constant (2 mM) and variable (10–500 mM), respectively. Michaelis-Menten parameters were determined by Lineweaver-Burk plots. ND indicates the case in which the data could not be fitted to Lineweaver-Burk plots.

**Figure 2:**
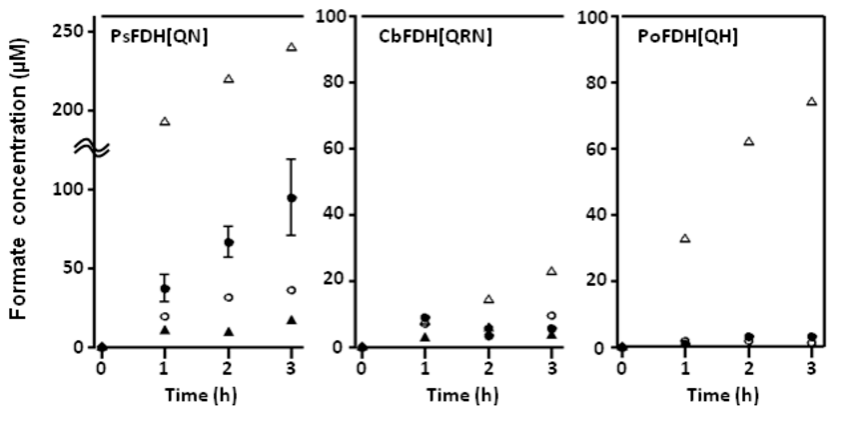
Formate production activities of NADPH-FDHs. The reaction solution contained 1.67 mg/mL of FDH, 1 mM NAD(P) H, 0.05% Triton X-100 and 1.1–1.3 mg/ml dissolved CO_2_. The concentration of formate was determined by a HPLC equipped with a Shim-pack SCR-102H column and a conductivity detector, CDD-10A_VP_ (Shimadzu, Kyoto, Japan). Open circles, closed circles, open triangles and closed triangles represent data of combinations of WTs with NADPH, mutants with NADPH, WTs with NADH, and mutants with NADH, respectively. All measurements were performed in triplicate.

**Figure 3:**
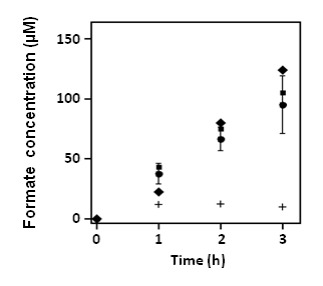
Substrate dependence and light sensitivity of PsFDH(QN). The formate production in the presence of 1 mM NADPH (circles) were compared with that in the presence of 10 mM NADPH (diamonds), that under visible light at an intensity of 1000 µmol photon m^-2^ s^-1^ (squares), and that in which NaHCO_3_ was used instead of CO_2_ (crosses). These experiments were performed as mentioned in Figure 2, except as described above.

Cofactor specificities of PsFDH(QN), CbFDH(QRN), and PoFDH(QH) for formate production were determined for the following reaction: NAD(P)H + CO_2_ → HCOO^-^ + NAD(P)^+^. Because this reaction is inhibited by gaseous oxygen, it was performed under strict anaerobic conditions (100% CO_2_ atmosphere). Consequently, we observed that the reactivity of PsFDH(QN) with NADPH was 5 times that with NADH, and produced formate at 32 µM/h, corresponding to a specific activity of 19 nmol (mg protein)^-1^ h^-1^, which was twice the amount produced in the reaction of the wild-type protein with NADPH (Figure 2). The reaction of PsFDH(QN) was also carried out in the presence of a high concentration of NADPH (10 mM), but it resulted in only a 1.3-fold activity compared to when 1 mM NADPH was used (closed diamonds in Figure 3). This suggested that the cofactor binding sites were almost saturated with NADPH at 1 mM concentration.

To confirm the photosensitivity of PsFDH(QN), the reaction was conducted under visible light (<420 nm) at an intensity of 1000 µmol photon m^-2^ s^-1^. No significant change was observed, demonstrating an insensitivity to light (closed squares in Figure 3). When instead of charging with CO_2_, NaHCO_3_ was added to the phosphate buffer (final concentration 50 mM) with NADPH, and Triton X-100, followed by degassing and mixing with the FDH solution, the rate of formate production was significantly lower (crosses in Figure 3). The observed concentration of dissolved CO_2_ in the buffer (0.040–0.060 mg/mL) was significantly lower than that in the solution charged with CO_2_ (1.1–1.3 mg/mL), which explains why no significant formate production from NaHCO_3_ was observed. The significant low CO_2_ concentration in the buffer added with NaHCO_3_ was mainly attributed to the degassing process.

CbFDH(QRN) and PoFDH(QH) showed much lower formate production activities than PsFDH(QN) (2.4 µM/h and 1.3 µM/h [1.4 and 0.77 nmol (mg protein)^-1^ h^-1^], respectively), likely due to their tendency to aggregate and become inactive (Figure 2).

### In vitro light-driven formate production

**Figure 4:**
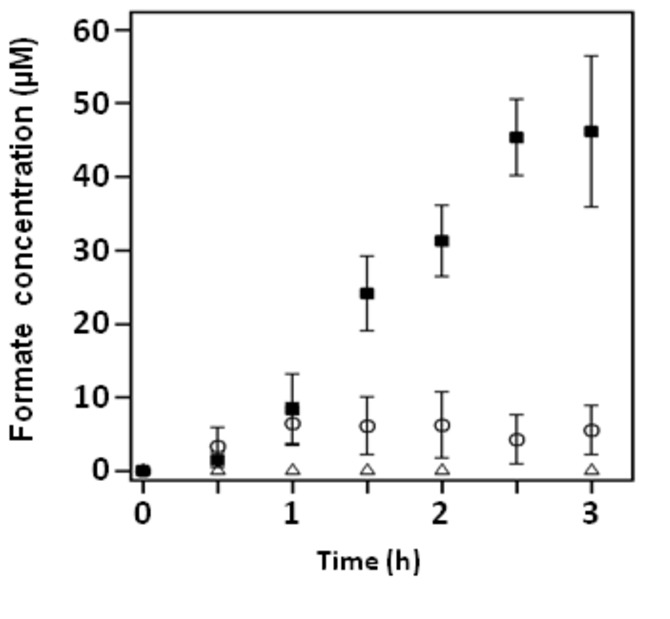
*In vitro* light-driven CO_2_ fixation by PS I and NADPH-FDH. The reaction solutions contained PS I (100 µg chlorophyll/mL), 10 mM ascorbate, 10 µM PC, 2 µM Fd, 0.5 µM FNR, 1 mM NADP^+^, and 4 mM NADPH, 2.5 mg/mL (~56 µM) PsFDH(QN), 0.05% Triton X-100 and 10% sucrose in PBS. The solution were degassed and then charging with 100% CO_2_ for 1 min. The reaction was initiated by illuminating with visible light (<420 nm) at an intensity of 1000 µmol photon m^-2^ s^-1^, and monitored by sampling at regular intervals without exposure to O_2_ (closed square). The control reactions were performed without the illumination (open circles) and in the presence of 10% oxygen gas (open triangles).

Using PsFDH(QN), a light-driven formate production system was constructed *in vitro*. The system consisted of PS I (100 µg chlorophyll/mL) as photosensitizer; 10 mM ascorbate as a sacrificial electron donor; 10 µM PC as an electron carrier at the donor side of PS I; 2 µM Fd, 0.5 µM FNR, 1 mM NADP^+^, and 4 mM NADPH as an electron relay system at the acceptor side of PS I; and 2.5 mg/mL (~56 µM) PsFDH(QN) as the catalyst for formate production. The formate concentration linearly increased with illumination time after about a 30-min lag time (closed squares in Figure 4). The maximum rate of formate production achieved was 26 µM/h. When expressed in units of formate per mg chlorophyll per hour, the rate is equivalent to 0.26 µmol (mg chlorophyll)^-1^ h^-1^. In the control reaction under dark condition (open circles in Figure 4), formate production was observed at a rate of 5 µM/h, which was significantly lower than that under illumination. In the reaction under a 10% O_2_ and 90% CO_2_ gas atmosphere (open triangles in Figure 4), formate was not detected, demonstrating the O_2_-sensitivity of the system.

The rate-determining factor was then assessed using the total activity of PsFDH(QN) and the rate of supply of NADPH. The total activity of 7.5 mg of PsFDH(QN) in 3 mL of reaction solution was estimated to produce 47 µM formate/h using its specific activity of 19 nmol (mg protein)^-1^ h^-1^ as described above. This rate was in the same range as the maximum rate of formate production at 30-60 min after initiation. On the other hand, the total activity of the 0.3 mg chlorophyll PS I in 3 mL solution was estimated to be 15 mM NADPH/h using its specific activity of 150 µmol (mg chlorophyll)^-1^ h^-1^ which was determined in the same conditions except for no FDH protein and no NADP^+^ being added and the concentration of PS I being 5 µg chlorophyll/mL. However, because the total NADP(H) concentration was 5 mM, almost all NADP^+^ was expected to be reduced to NADPH. In fact, the NADPH concentration in the control reaction solution without PsFDH(QN) was analyzed spectrophotometrically and estimated to reach 4.5 mM (NADPH/NADP^+^ ~ 9), which is high enough to push the equilibrium towards formate production, within 60 min after the initiation of the reaction under anaerobic conditions. The time required for all NADP^+^ to be converted to NADPH was consistent with the lag time observed in Figure 4. Therefore, the rate-determining factor was identified in the accumulation of NADPH from 0 min to 30 min, and in the activity of FDH from 30 min onward.

### In vivo light-driven formate production

To recreate the light-driven formate production system *in vivo*, we attempted to express PsFDH(QN) in *Anabaena* heterocysts, which differentiate in order to carry out nitrogen fixation during nitrogen starvation [10]. Heterocysts are deficient in PS II, and their internal environment is micro-oxic. The cells receive carbohydrates from neighboring vegetative cells and use them as electron donors to PS I. The photo-excited electrons derived from PS I are used for the reduction of N_2_ gas to ammonium by nitrogenase [20]. The micro-oxic and reductive environment makes heterocysts an ideal reaction vessel for light-driven formate production by PsFDH(QN). For the expression of PsFDH(QN) in heterocysts, we constructed two shuttle vectors, pAM505-fdh and pAM505-fdhfnr, and introduced them into *Anabaena* ( *Anabaena* -fdh and *Anabaena* -fdhfnr).

**Figure 5:**
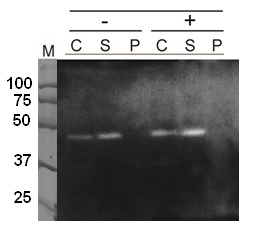
Expression of PsFDH[QN] in *Anabaena* -fdh. *Anabaena* -fdh cells were cultured in non-inducible ((-), BG11) or inducible ((+), BG11_0_) medium and their whole lysates (C), lysate supernatants (S) and lysate precipitates (P) were analyzed by Western blotting using anti-Histag antibody HRP conjugate. Chemiluminescent signals were detected at approximately 44 kDa as expected.

To confirm the expression of PsFDH(QN), western blot analysis was carried out. *Anabaena* -fdh and *Anabaena* -fdhfnr were cultivated in nitrogen-deficient BG11 medium (BG11_0_), in which the differentiation to heterocysts and the expression of PsFDH(QN) was to be induced. Cell extracts were separated on 10% polyacrylamide gels and then blotted onto polyvinylidene difluoride membranes. Chemiluminescence images were obtained after blocking, incubation with anti-His tag antibody-biotin conjugate and streptavidin-Horseradish Peroxidase conjugate and reaction with chemiluminescence reagent. The bands were successfully detected at the expected molecular weight of PsFDH(QN) in the samples from both exoconjugants, but the intensity of the sample from BG11_0_ was approximately 2-fold that from BG11 (Figure 5). Although it is suggested that the expression of the PsFDH(QN) was enhanced in response to nitrogen deprivation, it remains to be solved whether the increase occurred mainly in the heterocysts. Assuming the heterocyst-specific response, taken together with the fact that approximately 1 heterocyst develops for every 10–20 vegetative cells; it is speculated that the PsFDH(QN) concentration in the heterocysts was 10–20 times that in the vegetative cells.

**Figure 6:**
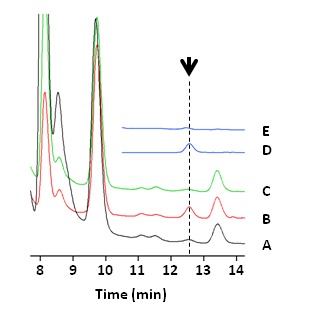
Formate production by pAM505-fdh exoconjugant cyanobacteria. The wild-type and exoconjugant *Anabaena* cultures (BG11, OD_700_ ~3) were inoculated into BG11_0_ and cultivated for 36 h under a light of 15 µmol photon m^-2^ s^-1^ and under air atmosphere. The media were then degassed and bubbled with argon + 10% CO_2_ for 12 h under a light of 150 µmol photon m^-2^ s^-1^. The cells anaerobically harvested were homogenized in 1N HCl. The resulting lysate was analyzed by ion-chromatography and the chromatograms of *Anabaena* -WT (A) and *Anabaena* -fdh (B) are shown. The lysate of *Anabaena* -fdh was also treated with *Candida* *boidinii* FDH (Sigma-Aldrich, Missouri, USA) and NAD^+^ overnight and was then analyzed (C). The chromatograms of formate solutions without and with the treatment with *Candida* *boidinii* FDH and NAD^+^ are shown as controls (D and E, respectively).

*Anabaena* -WT, *Anabaena* -fdh and *Anabaena* -fdhfnr were initially grown in BG11_0_ medium by bubbling with air, air + 10% CO_2_, N_2_ + 10% CO_2_, or argon + 10% CO_2_. We observed that only when *Anabaena* -fdh was incubated under argon + 10% CO_2_, a peak was detected at the same retention time (12.6 min) as formate with an intensity corresponding to a concentration of 28 µM (Figure 6). To confirm whether the peak could be assigned to formate, the lysate was neutralized and treated with 10 µM *Candida* *boidinii* FDH (Sigma-Aldrich, St. Louis, Missouri) and 10 mM NAD^+^ to advance the reaction: HCOO^-^ + NAD^+^ → CO_2_ + NADH. Thereupon, only the peak at 12.6 min among the observed peaks disappeared (Figure 5), demonstrating that it was derived from formate. Therefore, we concluded that introducing PsFDH(QN) into heterocysts accelerated *in vivo* formate production.

One of the possible interpretations of this result is that under argon + 10% CO_2_, the electron flux toward N_2_ fixation in heterocysts is decreased and the NADPH concentration is increased, facilitating the reaction: CO_2_ + NADPH → HCOO^-^ + NADP^+^ by PsFDH(QN). Another interpretation is that nitrogenase can accept electrons in the absence of N_2_ and produce H_2_ [20], which reacts with endogenous hydrogenase, producing NAD(P) H, which is used as a substrate for PsFDH(QN) to produce formate. In this study, no significant amounts of formate were detected in the lysates of *Anabaena* -fdhfnr. Although the level of expression of PsFDH(QN) in *Anabaena* -fdhfnr was almost the same as that in *Anabaena* -fdh, the growth rate of *Anabaena* -fdhfnr was lower than *Anabaena* -WT and *Anabaena* -fdh (see Figure S1), and a large quantities of cell debris appeared in the BG11_0_ medium, suggesting the occurrence of cell lysis and the outflow of cell contents. Notably, by over repeated subculture, the ability of *Anabaena* -fdh to produce formate sometime disappeared because of the appearance of plasmids lacking FDH gene. In addition to this problem, the analysis of these phenomena at molecular level becomes difficult because of the low frequency of heterocysts. The development of stable expression system and a quantitative understanding of the process in heterocysts are currently under investigation.

## Summary

Here we have shown a novel example of direct CO_2_ reduction using the photo-reducing power of PS I. The system proposed here consisted of proteins (PS I, Fd, FNR, and PsFDH[QN]) and a natural electron carrier (NADP[H]). We have demonstrated the feasibility of creating a CO_2_ reduction system *in vivo* by introducing PsFDH(QN) into heterocysts and have successfully enhanced the level of formate production. Further work investigating the detailed electron flux in the cells needs to be carried out, in addition to the optimization of the electron supply to the CO_2_ reduction system, the suppression of the N_2_ fixation and the design of the system for formate secretion.

## Supporting Information

Figure S1 **Growth curves of *Anabaena* wild-type (open circles), *Anabaena* -fdh (closed squares) and *Anabaena* -fdhfnr (closed triangles).** xmlns:xlink="http://www.w3.org/1999/xlink" xmlns:mml="http://www.w3.org/1998/Math/MathML">Culture conditions were: at 30^◦^C, light intensity of 30 mE∙m^-2^∙ s^-1^ and bubbling with air in BG11 medium.
(TIF)
